# Epidemiology and fitness effects of wood mouse herpesvirus in a natural host population

**DOI:** 10.1099/vir.0.044826-0

**Published:** 2012-11

**Authors:** Sarah C. L. Knowles, Andy Fenton, Amy B. Pedersen

**Affiliations:** 1 Centre for Immunity, Infection and Evolution (CIIE), Institute of Evolutionary Biology, School of Biological Sciences, The University of Edinburgh, West Mains Road, Edinburgh, EH9 3JT, UK; 2 Institute of Integrative Biology, University of Liverpool, Biosciences Building, Crown Street, Liverpool, L69 7ZB, UK

## Abstract

Rodent gammaherpesviruses have become important models for understanding human herpesvirus diseases. In particular, interactions between murid herpesvirus 4 and *Mus musculus* (a non-natural host species) have been extensively studied under controlled laboratory conditions. However, several fundamental aspects of murine gammaherpesvirus biology are not well understood, including how these viruses are transmitted from host to host, and their impacts on host fitness under natural conditions. Here, we investigate the epidemiology of a gammaherpesvirus in free-living wood mice (*Apodemus sylvaticus*) and bank voles (*Myodes glareolus*) in a 2-year longitudinal study. Wood mouse herpesvirus (WMHV) was the only herpesvirus detected and occurred frequently in wood mice and also less commonly in bank voles. Strikingly, WMHV infection probability was highest in reproductively active, heavy male mice. Infection risk also showed a repeatable seasonal pattern, peaking in spring and declining through the summer. We show that this seasonal decline can be at least partly attributed to reduced recapture of WMHV-infected adults. These results suggest that male reproductive behaviours could provide an important natural route of transmission for these viruses. They also suggest that gammaherpesvirus infection may have significant detrimental effects in wild hosts, questioning the view that these viruses have limited impacts in natural, co-evolved host species.

## Introduction

Gammaherpesviruses are a widespread group of DNA viruses that form characteristically persistent infections in a range of mammal species (Davison, 2002). Since human gammaherpesviruses are largely host specific, murine gammaherpesviruses have become important models for studying fundamental aspects of virus–host interactions. In particular, the pathogenesis and immune interactions of murid herpesvirus 4 (MuHV-4, of which MHV-68 is the archetypal strain) have been well-studied in inbred laboratory mice (Sunil-Chandra *et al.*, 1992; Simas & Efstathiou, 1998).

For all murine gammaherpesviruses, the natural route of transmission is not well understood. In the laboratory, experimental inoculations of MuHV-4 are generally performed intranasally. Since the intranasal route is the most infectious, the nasal mucosa is considered the most likely natural point of viral entry (Milho *et al.*, 2009). However, laboratory experiments where naïve animals have been exposed to MuHV-4-infected mice in the same cage have failed to produce new infections, suggesting that transmission may not occur via a simple respiratory route (François *et al.*, 2010). Several natural mouse behaviours are curtailed under laboratory conditions, including scent marking, nesting, aggressiveness and sexual interactions, since experiments generally involve only female mice. The lack of transmission in a cage suggests that such interactions may be important in natural gammaherpesvirus transmission. How these viruses enter their host influences several important aspects of infection, including the extent to which different cell types are involved in lytic replication and long-term latency (Sunil-Chandra *et al.*, 1992; Weck *et al.*, 1996), and the location and intensity of initial MuHV-4 replication (Milho *et al.*, 2009; François *et al.*, 2010). Thus, elucidating the natural transmission route of murine gammaherpesviruses remains a key goal and is necessary to ensure that inoculation under controlled laboratory conditions mimics natural infection as closely as possible.

It is also recognized that murine gammaherpesviruses can behave differently in natural and non-natural host species. Although the investigation of MuHV-4 in laboratory mice has yielded many important insights, *Mus musculus* is not known to be a natural host for this virus, which was originally isolated from a bank vole (*Myodes glareolus*) (Blaskovic *et al.*, 1980) but can also naturally infect yellow-necked field mice (*Apodemus flavicollis*) and, more rarely, wood mice (*Apodemus sylvaticus*) (Ehlers *et al.*, 2007). Several aspects of disease vary between natural and model hosts, including the extent of lytic replication in the lungs, lung histopathology, the precise site of latency and antibody responses (Hughes *et al.*, 2010a; François *et al.*, 2010). Thus, studies of murine gammaherpesviruses in their natural host species constitute an important check on biological conclusions reached in laboratory mice and are important for understanding the natural epidemiology and aetiology of these viruses.

Most studies of herpesviruses in wild rodents to date have been surveys (Ehlers *et al.*, 2007, 2008; Hughes *et al.*, 2010b) and, to our knowledge, only one published epidemiological study of murine gammaherpesviruses exists (Telfer *et al.*, 2007). This serological study suggested that MuHV-4 was common in wood mice (and to a lesser extent bank voles) in the UK, and that heavy males had the highest seroprevalence. However, infection was diagnosed based on antibody presence alone (Blasdell *et al.*, 2003; Telfer *et al.*, 2007) and since antibodies against different gammaherpesviruses may be cross-reactive, exactly which virus or viruses were investigated remains unclear. A subsequent study sequenced eight herpesvirus isolates from wood mice in the same geographical area and identified all of them as a novel virus distinct from MuHV-4, wood mouse herpesvirus (WMHV; Hughes *et al.*, 2010b), which has recently been assigned the species name MuHV-7. Overall, uncertainty remains about which gammaherpesviruses naturally infect wood mice and bank voles, and to what extent previous sero-epidemiological findings relate to either MuHV-4 or WMHV.

Studies of gammaherpesviruses in wild rodents also provide an opportunity to examine the potential fitness costs of these viruses in natural hosts. Gammaherpesviruses have been suggested to be benign, due to prolonged host–virus co-evolution (Davison, 2002; Ackermann, 2006). However, virulence theory indicates that co-evolution does not necessarily give rise to pathogens of low virulence, and that, depending on a multitude of factors, virtually any level of virulence can evolve (Anderson & May, 1982; Frank, 1996). Despite the relatively well-documented pathogenicity of gammaherpesviruses in humans and domesticated animals (Ackermann, 2006; Fortier *et al.*, 2010), their impacts in wild animals have not been explored. Since survival of wild rodents can be examined by monitoring host recapture over time, they provide an ideal opportunity to test the potential fitness consequences of gammaherpesviruses in a natural transmission setting.

Here we investigate the epidemiology of herpesvirus infection over a 2-year period in wild wood mice and bank voles in the UK. We first show through sequencing that only WMHV is found in these wild populations, and then explore ecological and host predictors of WMHV infection in wood mice to shed light on its natural transmission route. Finally, by analysing the relationship between infection and mouse recapture, we investigate whether infection could affect host survival in a wild setting.

## Results

### Herpesvirus identity

The number of samples screened and sequences obtained with each PCR method are shown in Table S1 (available in JGV Online). Using a pan-herpesvirus PCR, DPOL sequences (160 bp excluding primers) were obtained from eight wood mice and three bank voles. In both host species, all sequences were identical to the genome sequence of WMHV (GenBank accession no. GQ169129.1). Of the 68 sequences obtained from wood mice (*n* = 26 in 2009 and *n* = 42 in 2010) using the non-degenerate PCR (which is capable of detecting both WMHV and MuHV-4; see Methods), all were identical to WMHV across the 160 bp length.

### Species difference in WMHV prevalence

Table 1 shows the number of animals of each species screened for infection by each method per year. We found evidence for a marked species difference in WMHV prevalence; in 2010, where both species were systematically screened, seroprevalence was approximately three times higher in wood mice than in bank voles (22.5 versus 6.1 % across all samples, *n* = 742 and *n* = 164, respectively). Due to low prevalence and less systematic screening among bank voles, subsequent analyses to compare diagnostic methods and test predictors of infection involved only wood mice.

### Comparison of diagnostic methods and detection of maternal antibodies

**Fig. 1.**
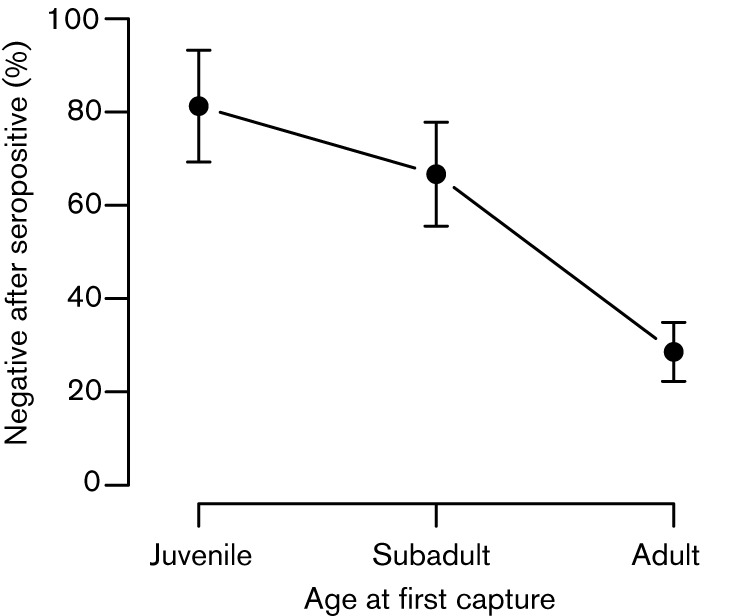
Relationship between host age and the proportion of times that a WMHV-seropositive mouse was subsequently seronegative. Predicted values from a binomial GLM are plotted, with error bars indicating sem.

A total of 828 samples (from 466 individual mice caught in 2009; Table 1) were diagnosed with both the non-degenerate PCR assay and an immunofluorescence assay (IFA). Overall, the agreement in infection status between these methods was high (χ^2^_1_ = 77.86, *P*<0.0001). The IFA detected antibodies in 63 % of PCR-positive samples, while PCR detected viral DNA in 22 % of seropositive samples. In datasets containing only one data point per mouse, adults showed a strong and significant agreement between diagnostic methods, whereas subadults and juveniles did not (Fisher’s exact test, adults *P*<0.0001, *n* = 341; subadults *P* = 0.228, *n* = 161; juveniles *P* = 1.00, *n* = 81). Specifically, 23 % of seropositive adults were also positive by PCR, whereas only 10 % of seropositive subadults and 0 % of juveniles (0/8 individuals) were also positive by PCR. This suggests that seropositivity in adults probably reflects actual WMHV infection, whereas in subadults and juveniles it may often reflect maternal antibodies. Serological histories from recaptured individuals showed that, once infected, antibodies were not always detectable at successive time points: 70 of 175 (40 %) samples from mice that were seropositive at a previous capture were seronegative. Supporting the existence of maternal antibodies, the propensity for WMHV antibodies present at first capture to disappear over time depended on age: juveniles and subadults that had WMHV antibodies at first capture had a higher proportion of subsequent seronegative captures than adults (age: χ^2^_2_ = 15.10, *P* = 0.001, *n* = 56; Fig. 1) and a similar negative relationship was seen with body length, a finer scale measure of age (χ^2^_1_ = 23.07, *P*<0.001, *n* = 56). This within-individual effect was also visible in cross-sectional, population-level analyses. Across juveniles and subadults, the relationship between body length and seropositivity was flat or significantly negative (juveniles χ^2^_1_ = 4.63, *P* = 0.031, *n* = 110; subadults χ^2^_1_ = 0.18, *P* = 0.674, *n* = 322), whereas in adults it was strongly positive (χ^2^_1_ = 39.20, *P*<0.001, *n* = 673). These differences manifest as a significant body length*age interaction in a generalized linear model (GLM) including all age categories (Fig. S1, available in JGV Online; body length*age χ^2^_2_ = 14.49, *P* = 0.001, *n* = 899). Such an interaction is not seen for PCR positivity (body length*age χ^2^_2_ = 0.00, *P* = 0.998, *n* = 466, Fig. S1), indicating that it is driven by differences in the presence of maternal antibodies. Although our results suggest that IFA positivity often reflected maternal antibodies in young animals, they also indicate that IFA was more sensitive at detecting infection than PCR: in adults (where maternal antibodies are negligible), PCR detected DNA in only 22 % of IFA positive samples.

**Table 1.** Summary of samples and individuals systematically screened for herpesvirus prevalence using either IFA, or IFA and the non-degenerate nested PCR

	No. of samples (individuals*) screened by IFA			No. of samples (individuals*) screened by non-degenerate PCR and IFA		Consensus WMHV prevalence both years (95 % CI)
	2009	2010	Both years	2009	2010	
Total wood mouse (*A. sylvaticus*)	921 (479)	742 (420)	1663 (899)	828 (466)	0 (0)	0.176 (0.151, 0.201)
Adults	660 (352)	551 (321)	1211 (673)	575 (341)	0 (0)	0.211 (0.180, 0.242)
Subadults	179 (168)	163 (154)	342 (322)	161 (161)	0 (0)	0.084 (0.054, 0.114)
Juveniles	82 (82)	28 (82)	110 (110)	81 (81)	0 (0)	0.145 (0.080, 0.211)
Males	518 (274)	461 (263)	979 (537)	474 (268)	0 (0)	0.227 (0.192, 0.263)
Females	403 (205)	281 (157)	684 (362)	354 (198)	0 (0)	0.099 (0.069, 0.130)
Total bank vole (*M. glareolus*)	70 (64)†	164 (101)	234 (164)	0 (0)	0 (0)	

* Indicates dataset size when one data point was selected per individual in this category (to avoid pseudoreplication). WMHV prevalence for each group was calculated using a consensus diagnosis from both IFA and PCR data as described in the text, using datasets containing one data point per individual.

† In 2009, blood samples were taken from voles captured in June–July only and screened using IFA, rather than June–December as for mice in 2009. In 2010, blood samples were taken from all mice and voles captured in May–December.

### Predictors of WMHV infection in wood mice

**Fig. 2.**
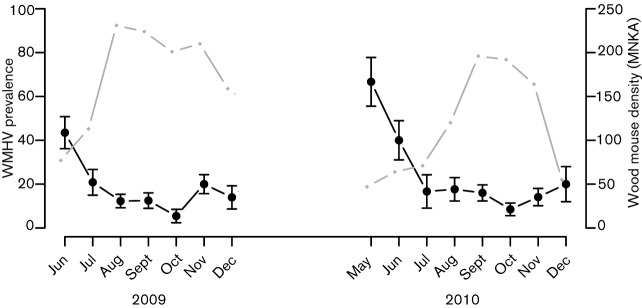
Seasonal changes in WMHV prevalence in wood mice (left axis, black points) and host density (right axis, grey points) during 2009 and 2010. WMHV infection status was taken as the consensus diagnosis from PCR and IFA, and host density is represented as the minimum number of wood mice known alive (MNKA) across all trapping grids. Raw data are plotted with sem for monthly prevalence estimates.

**Fig. 3.**
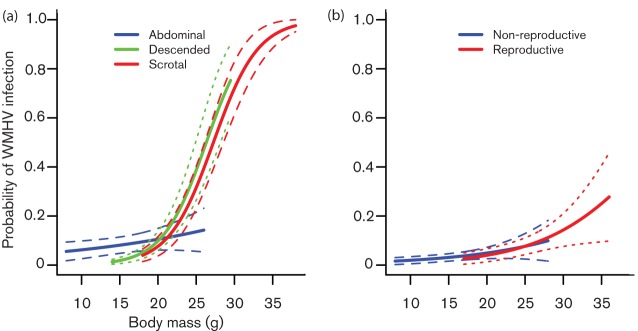
Effect of body mass on the probability of WMHV infection in male (a) and female (b) wood mice, according to reproductive status. Data are predicted values+sem from the minimal model in Table 2(a) (predicted values are for mice caught in August on grid MW1). Data are only plotted for the body mass range observed within each reproductive category.

Across both years, 17.6 % of wood mice were WMHV-positive, using a consensus measure of PCR and IFA diagnoses (Table 1). We identified three significant predictors of WMHV infection in wood mice: month, trapping grid and an interaction between body mass and reproductive status/sex (Table 2a). Infection prevalence varied seasonally each year, decreasing between spring and summer and then remaining relatively low until December (Fig. 2, Table 2a). Although an effect of age per se was not supported, an interaction between body mass (which is positively correlated with age) and reproductive status/sex was significant (Table 2a, Fig. 3). Using body length instead of body mass as a proxy for age gave an interaction of the same form and magnitude (χ^2^_1_ = 17.55, *P* = 0.002). The main difference in WMHV infection probability was between reproductive males (those classed as having descended or scrotal testes) and other animals (non-reproductive males and females), and became stronger among heavier mice (Fig. 3). Indeed, the interaction between body mass and a binary variable indicating whether an animal is a reproductively active male or not, was stronger than that between body mass and reproductive status/sex (body mass*reproductive male: χ^2^_1_ = 25.08, *P*<0.001; body mass*reproductive status/sex: χ^2^_4_ = 15.06, *P* = 0.005; Table 2a).

**Table 2.** Predictors of WMHV infection in wild wood mice in the UK Full models were simplified by backwards stepwise elimination. For variables not in the minimal model, statistics are those at the point that factor left the model. na, Indicates that significance of main effects is not reported, as interactions involving these terms remain in the minimal model.

Variable	df	Denominator df	χ^2^	*P*
**(a) All animals (*n* = 899)**				
Year	1	873	0.20	0.6586
Month†	7	877	35.48	<0.0001†
Month*year	6	866	3.76	0.7088
Sex/reprod†	4	877	na	na
Body mass†	1	877	na	na
Ticks	1	876	1.40	0.2364
Fleas	1	872	0.04	0.8526
Age	2	874	1.47	0.4786
Grid†	5	877	14.47	0.0129†
Sex/reprod*body mass†	4	877	15.06	0.0046†
**(b) Males only (*n* = 537)**				
Year	1	515	0.29	0.5913
Month†	7	524	23.06	0.0017†
Month*year	6	508	3.58	0.7329
Reprod†	2	524	na	na
Body mass†	1	524	na	na
Ticks	1	523	1.14	0.2861
Fleas	1	514	0.03	0.8530
Age	2	516	0.94	0.6263
Grid	5	518	7.33	0.1974
Reprod*body mass†	2	524	10.68	0.0048†
**(c) Females only (*n* = 362)**				
Year	1	344	0.27	0.6064
Month†	7	353	21.08	0.0037†
Month*year	6	336	4.87	0.5610
Reprod	4	342	0.00	0.9559
Body mass†	1	353	7.21	0.0072†
Ticks	1	343	0.39	0.5339
Fleas	1	347	1.76	0.1848
Age	2	345	1.56	0.4580
Grid	5	348	10.68	0.0582
Reprod*body mass	1	335	0.01	0.9274

† Variables in the minimal model (*P*<0.05).

To better understand the roles of host sex, reproductive activity and body mass for WHMV infection, we reran the analyses for males and females separately. For males, there was a clear interaction between body mass and reproductive status; the probability of WMHV infection only increased strongly with body mass among reproductively active males (Fig. 3, Table 2b). In contrast, females showed only a weak effect of body mass, and no evidence for any effect of reproductive status (Table 2c). In models including only adults (where maternal antibodies should be negligible), findings were very similar (Table S2, Fig. S2). Among 486 initially WMHV-negative mice, 39 became infected by the following month. The probability of becoming infected did not vary according to month or reproductive status, and was only predicted by host age, with juveniles less likely to become infected than adults or subadults (Table S4).

### WMHV infection and mouse recapture

Since murine gammaherpesvirus infections are thought to be lifelong, the observed seasonal decline in prevalence could be caused by one of several non-mutually exclusive processes: (i) a seasonal decline in infection detectability, (ii) a seasonal influx of uninfected individuals, (iii) increased survival over winter of WMHV-infected individuals or (iv) the selective loss of infected individuals. We examined all possibilities. From longitudinal data, we found no evidence that the initial capture date (as a linear predictor) influenced the proportion of times a mouse that was seropositive at first capture was subsequently seronegative, while controlling for host age (date: χ^2^_1_ = 0.17, *P* = 0.678, age: χ^2^_2_ = 6.12, *P* = 0.004), suggesting no seasonal variation in antibody disappearance. It is also unlikely that the annual influx of newborn animals plays a major role in this seasonal decline, as population density increased and the young of the year only became adults after the seasonal decline in WMHV prevalence had occurred (Figs 2 and S3). Furthermore, the decline in prevalence was observed when analysis was restricted to adults that were not young of the year (Fig. S3), and among all adults when body length (as a fine-scale measure of age) was controlled for (month χ^2^_7_ = 25.08, *P*<0.001, body length χ^2^_1_ = 0.92, *P* = 0.338). However, we did find evidence that recapture duration for WMHV-infected adults was on average half a month shorter than for uninfected adults, i.e. 25 % lower than WMHV-negative adults within a given year (Table 3). To further explore whether other factors associated with WMHV infection (e.g. reproductive status, sex and size-related variables) could drive this recapture effect, we tested several of these within the minimal model, but found that none could explain the recapture effect as well as WMHV infection (Table S5). Since it is possible that high WMHV prevalence in spring could be partially driven by increased survival over winter of infected individuals, we tested for this possibility, but found no evidence for such an effect. Survival over winter of mice from 2009 to spring 2010 was strongly predicted by the month in which they were first caught, but not by whether they were ever WMHV positive in 2009, or an interaction between these two factors (month χ^2^_6_ = 30.90, *P*<0.001, WMHV χ^2^_1_ = 0.00, *P* = 0.961, month*WMHV χ^2^_6_ = 5.38, *P* = 0.496, *n* = 520).

**Table 3.** Predictors of host recapture duration (months between first and last capture) in wild adult wood mice Models were simplified by backwards stepwise elimination. For variables not in the minimal model, statistics are those at the point that factor left the model. na, Indicates that significance of main effects is not reported, as interactions involving these terms remain in the minimal model.

Variable	df	Denominator df	χ^2^	*P*
Grid	5	395	3.21	0.668
Month	5	402	na	na
Year	1	402	na	na
Month*year†	5	402	11.28	0.046†
Reproductive male	1	401	1.13	0.288
Body mass	1	400	1.27	0.260
Reproductive male*body mass	1	393	0.00	0.960
WMHV†	1	402	4.71	0.030†
WMHV*year	1	394	0.41	0.522

† Variables in the minimal model (*P*<0.05).

## Discussion

Serological surveys of wild rodents in the UK have reported that MuHV-4, the major laboratory model of murine gammaherpesvirus infection, is prevalent in wild wood mice and bank voles (Blasdell *et al.*, 2003; Telfer *et al.*, 2007). Here, we show that the virus prevalent is WMHV rather than MuHV-4 by using sequences from 68 wood mice in the same geographical region (Cheshire). Our findings support those of Hughes *et al.* (2010b), who detected only WMHV sequences in a smaller sample of wood mice from the same region. Although MuHV-4 has been detected in wood mice from continental Europe (Ehlers *et al.*, 2007), taking together all three studies reporting sequence data on gammaherpesviruses from wood mice to date (present study; Ehlers *et al.*, 2007; Hughes *et al.*, 2010b), WMHV is the most common gammaherpesvirus in this host species. Given this, WMHV infection in *A. sylvaticus* could provide a useful laboratory model for investigating herpesvirus biology in a naturally occurring host–virus pair, particularly since the genome of WMHV is now available (Hughes *et al.*, 2010b). Such a model could be particularly useful given that interactions between herpesviruses and the host immune system are not always expressed in an artificial host species with which the virus has not co-evolved (Hughes *et al.*, 2011). We also detected WMHV in all three sequences obtained from bank voles (*M. glareolus*), which is the first record in this host species.

Of the two assays used to screen for herpesvirus, immunofluorescence-based antibody detection was more sensitive than PCR on peripheral blood. This may be because latently infected B cells are not as reliably present in peripheral blood as antibodies and may reside in lymphoid organs much of the time. However, we also found clear evidence for maternal antibodies to WMHV that persist into young adulthood, as is known to occur for other viruses of small rodents (Botten *et al.*, 2002; Kallio *et al.*, 2006). Thus, antibody detection is not a perfect indicator of herpesvirus infection in wild rodents and care should be taken to avoid inference about infection based on the presence of maternal antibodies.

The natural route of transmission for murine gammaherpesviruses has not been established, and attempts to instigate transmission among female laboratory mice in cages have failed (François *et al.*, 2010), suggesting that transmission does not occur through inhalation of airborne virus particles or general contact among individuals. Our analyses shed light on how these viruses may be transmitted in the wild, by showing that infection probability was strongly associated with reproductive status and sex. Reproductively active males were much more likely to be infected than non-reproductive males or females, particularly when they also had high body mass (Fig. 3). Telfer *et al.* (2007) previously showed that herpesvirus infection in wood mice (identified serologically as MuHV-4, though probably WMHV) was predicted by an interaction between body mass and sex. While we can detect the same interaction, crucially we show that it is not just that heavier males have a higher probability of WMHV infection, but specifically those that are reproductively active. In the heaviest, reproductively active males, the predicted probability of infection reaches almost 1 (Fig. 3), indicating that WMHV infection is strongly clustered in these individuals. This pattern suggests that male reproductive activity may increase exposure to WMHV. Purely sexual transmission seems unlikely, as in a promiscuous species like the wood mouse (Booth *et al.*, 2007), theory predicts that higher variance in mating success among males would drive a female bias in prevalence for a sexually transmitted infection (Thrall *et al.*, 2000; Gouveia-Oliveira & Pedersen, 2009). Our results suggest that specific types of interactions occurring predominantly between reproductive males, such as biting or territorial scent-marking behaviour, may form an important transmission route. Interestingly, the epidemiological patterns of WMHV infection reported here are similar to those seen for hantaviruses, in that infection probability is highest for heavy adult males (Mills *et al.*, 1997; Bennett *et al.*, 1999; Douglass *et al.*, 2001). A number of studies have shown that hantavirus infection probability is increased in males with wounds or scarring, suggesting that biting plays a role in hantavirus transmission (Mills *et al.*, 1997; Douglass *et al.*, 2001; Hinson *et al.*, 2004; Calisher *et al.*, 2007). Biting could similarly play a role in murine gammaherpesvirus transmission, with the virus being transmitted through saliva. However, scent-marking behaviour is perhaps a better candidate for the natural transmission route, whereby mice inhale viral particles from the urine of other individuals when demarcating territories. The nose is considered the most likely point of viral entry for MuHV-4, as intranasal inoculation of laboratory mice is highly effective at establishing infection (Milho *et al.*, 2009). Although, as with many viruses, MuHV-4 is shed in several body fluids (e.g. urine, saliva, milk), it has been shown that intranasal infection leads to higher levels of urinary viral excretion than other inoculation routes (Hricová & Mistríková, 2008), consistent with transmission via scent-marking behaviour. It is also possible that reproductive males are no more exposed to infection than other individuals, but that the difference in prevalence is caused by differences in susceptibility and infectiousness, or both. Reproductive males may be more susceptible to infection, if for example, hormones such as testosterone increase susceptibility to infection (Folstad & Karter, 1992) or if they are in generally poorer condition. Although reproductive hormones are known to have diverse effects on immune responses (Klein, 2004), the evidence that hormonal changes associated with reproduction could increase susceptibility to infection in free-ranging populations is mixed at best (Roberts *et al.*, 2004; Lehmer *et al.*, 2010; Ezenwa *et al.*, 2012). It has, however, been shown for Seoul virus (a hantavirus) in Norway rats that older, heavier males not only show a higher prevalence of infection, but also shed more virus in body fluids than younger males (Hinson *et al.*, 2004). Thus, if reproductive males interact with one another more often than other types of individual, such increased viral shedding could contribute to a higher herpesvirus prevalence in heavy, reproductive males. Overall, these findings suggest that transmission experiments using reproductively mature males, both in single- and mixed-sex environments, may yield greater success in firmly establishing via which route transmission of murine gammaherpesviruses naturally occurs. Indeed, for Sin Nombre hantavirus, which shows similar ecological patterns to those reported here, natural transmission among cage mates was rare and only seen from male to male, but not among females or in mixed-sex groups (Botten *et al.*, 2002).

We found marked, repeatable, seasonal variation in WMHV prevalence. The increase in prevalence observed in both years between December and May suggests that the majority of transmission occurs during this period. During early spring, when male testes descend and females become reproductively receptive, the male home range size dramatically increases in wood mice (Randolph, 1977). Interactions among reproductive males may occur frequently during this time, when territories are being established. If male–male interactions are an important route of WMHV transmission, this could explain the observed spring peak in infection prevalence. Longitudinal data from individuals captured multiple times during summer/autumn did reveal the acquisition of some new infections during this time, though there was no indication that these transmission events were associated with reproductive status or sex. This could reflect reduced statistical power to detect the effects of reproductive status in this smaller dataset, or that reproductive behaviours are an important determinant of infection risk early on, but less so later in the year. For example, territory establishment at the start of the breeding season may facilitate transmission, but subsequent reproductive activity per se may pose less risk of infection. It could also be that the current reproductive condition becomes a poorer indicator of whether an individual has been reproductively active as the breeding season progresses, as males that have been reproductive return to a non-reproductive state. Despite some new infections being acquired in the summer and autumn, overall prevalence remains low during this period each year. Since the population density increases from July onwards, but WMHV prevalence remains low (Fig. 2), it appears that WMHV transmission is not density-dependent, as has been observed for another virus (cowpox) at one of our study sites (Telfer *et al.*, 2005).

On average, adults infected with WMHV were recaptured 25 % (or approximately half a month) less than uninfected individuals. Thus, contrary to suggestions that gammaherpesviruses cause virtually no disease in natural hosts (Davison, 2002; Ackermann, 2006), we find a negative association between WMHV infection and recapture duration among adult wood mice, suggesting that infection may reduce longevity in a natural host species. Exactly how WMHV infection might reduce the survival of wild wood mice is unknown. During latent infection, gammaherpesviruses are known to immunomodulate the host, by expressing a range of host immune gene mimics (McCormick & Ganem, 2005; Ackermann, 2006; Hughes *et al.*, 2011). Either this immunomodulation or latency-associated innate immune activation, as shown for MuHV-4 in laboratory mice (Barton *et al.*, 2007), could underlie the survival cost observed here. For example, although a state of heightened immune activation has been shown to protect against bacterial challenge and lethal lymphoma in laboratory mice (Barton *et al.*, 2007; White *et al.*, 2010), it could be costly in the wild. Immune activation may reduce resources available for other important physiological functions or cause immunopathology (Eraud *et al.*, 2005; Bertrand *et al.*, 2006; Hanssen, 2006). Interactions between latent WMHV and the host immune system could also reduce survival through carcinogenesis, as seen for Kaposi’s sarcoma-associated herpesvirus, or via interactions with other pathogens. Immune responses to other pathogens, of which there are many at our field sites (S. C. L. Knowles, A. B. Pedersen, A. Fenton & O. Petchey, unpublished data; Begon *et al.*, 1999; Behnke *et al.*, 1999; Birtles *et al.*, 2001), could be reduced in WMHV-infected individuals through impaired function of latently infected B-cells or by an altered cytokine/chemokine milieu within the host (Hughes *et al.*, 2011). Acute infection may also contribute to reduced recapture, through lung pathology (Hughes *et al.*, 2010a). However, although our results suggest that WMHV infection could reduce survival in wood mice, it is important to note these findings are correlational and do not distinguish differences in survival/emigration from differences in recapture probability. Thus alternative explanations for these findings remain possible. For example, it may be that individuals investing heavily in reproduction (e.g. large, reproductive males) have a shorter lifespan, or a larger home range such that they are less likely to be recaptured on our trapping grids.

In summary, we show that WMHV is a common infection in wild wood mice and that the probability of infection is highest among reproductively active, heavy males in the spring. These findings suggest that male reproductive behaviours could be an important component of natural murine gammaherpesvirus transmission and that use of male animals in laboratory- or enclosure-based studies may be useful to more fully understand how this virus is transmitted between, and subsequently exploits, its hosts. We also find a negative association between WMHV infection and host longevity. While it remains uncertain whether this association is causal, it raises the possibility that interactions between gammaherpesviruses and either the host immune system or other pathogens can have significant fitness consequences for wild hosts.

## Methods

### 

#### Field methods.

Wood mice (*A. sylvaticus*) and bank voles (*M. glareolus*) were trapped in 2009 and 2010 in two mixed woodlands in Cheshire, UK (two grids in Manor Wood, 53° 19′ N 3° 3′ W, and four grids in Haddon Wood, 53° 16′ N 3° 1′ W). One of these woodlands (Manor Wood) is the site that Telfer *et al.* (2007) used to investigate a virus identified serologically as MuHV-4 in the same two host species. On each grid, two live traps [H.B. Sherman 2×2.5×6.5 inch (5.08×6.35×16.51 cm) folding trap] baited with grain and bedding material were placed every 10 m in a 70×70 m square. Each grid was trapped monthly, for three consecutive nights during June–December in 2009 and May–December in 2010. All traps containing animals or evidence of animal activity were disinfected and autoclaved before reuse. All trapped animals were tagged using subcutaneous passive integrated transponder tags, so they could be individually identified upon recapture. For all wood mice in both 2009 and 2010, and for all bank voles in 2010, a blood sample was taken from the tail at first capture within a monthly trapping session. In 2009, bank voles were not systematically sampled, as blood was only taken from a small subset of voles caught in June and July. At each capture, several morphometric measurements were taken: animals were assigned to the following age groups: juvenile, subadult or adult, according to the pelage in the first instance, with body mass used as a secondary trait where pelage was inconclusive (under 12 g, juvenile; 12–16 g, subadult; over 16 g, adult); body length, mass, sex and reproductive status were recorded and the fur was brushed to record the presence of ticks and fleas. For males, reproductive status was assigned based on the testes’ position as either abdominal (testes not protruding), descended or scrotal. Female reproductive status was recorded as whether they were pregnant or not and whether the vagina was perforated. For the purposes of a different study, on five of the trapping grids mice were treated orally with either the anthelminthic drug Ivermectin (*n* = 448 mice, both years), the anti-coccidia drug Toltrazuril (*n* = 102, 2010 only) or a control solution of water (*n* = 173, both years). On the sixth grid, no treatments were applied in either year (*n* = 176). To confirm that these treatments did not affect our results, we repeated statistical analyses of ecological predictors of herpesvirus infection on the treated grids only, including treatment as a factor in the starting model to confirm that the use of these drugs did not affect patterns of herpesvirus infection. Results were virtually identical in these analyses, with treatment having no significant effect on infection probability (Table S3).

#### Immunological and molecular diagnosis of herpesvirus infection.

We used two methods to screen for herpesvirus infection. The first was a serological assay that detects antibodies in mouse serum using IFA and the second was a nested PCR assay, which we used to screen blood samples. A positive diagnosis for herpesvirus by serology may indicate either previous exposure to the virus or maternal antibodies in young individuals that have not encountered the virus. Conversely, since PCR positivity indicates the presence of viral DNA, individuals testing positive by PCR must harbour either acute- or latent-stage infections. Since murine gammaherpesviruses characteristically form latent infections, which are thought to be lifelong, seropositivity not resulting from maternal antibodies should reflect ongoing infection, and thus match PCR positivity.

#### IFA.

Where possible, blood samples were separated into serum for use in the IFA and packed cells for PCR screening. Where blood samples were too small to separate, whole blood was first tested in the IFA and subsequently, the same sample was used for the PCR assay. For the IFA, Vero cell monolayers in 96-well plates were inoculated with approximately 5 p.f.u. virus, fixed with 70 % ethanol after a 72 h incubation and stored until needed at 4 °C. The virus used to inoculate plates was isolated from the lung of an infected wood mouse captured in Hooton, Cheshire, UK, in July 1998. The identity of this virus isolate was confirmed by PCR and sequencing (see below) as WMHV. IFAs were performed by rehydrating the cells in PBS and making serial incubations with serum (diluted 1 : 20 and 1 : 40) and anti-mouse–FITC, and separating by washing in PBS, before viewing under UV illumination. The assay was previously optimized using sera from mice experimentally infected with MHV-68 (Telfer *et al.*, 2007) and serum from an MHV-68-infected laboratory mouse was used as a positive control. This assay is therefore known to detect both MuHV-4 and WMHV.

#### PCR and sequencing.

Genomic DNA was extracted using a protocol designed for small blood samples (Mackey *et al.*, 1998). To determine which herpesvirus was present in these samples, 82 wood mouse samples that were positive by IFA and all 64 bank vole samples (irrespective of IFA diagnosis) from 2009 were screened using the nested pan-herpesvirus PCR protocol of Ehlers *et al.* (2007). Since this degenerate nested PCR assay had very low specificity, a non-degenerate nested PCR assay was developed using the same regions of the DPOL gene for primer binding, but with primers exactly matching the WMHV genome sequence (GenBank acession no. GQ169129). Full details of PCR primers and conditions are given in the supplementary material. All blood samples from 2009 were screened using this non-degenerate PCR assay to compare sensitivity of this assay with that of the IFA. To confirm the identity of the virus detected with this non-degenerate PCR assay, all PCR products from 2009 (*n* = 26), as well as all IFA positives from 2010 that could be amplified (42 of 163 IFA positives), were directly sequenced in both directions. While the non-degenerate primers were designed based on a WMHV sequence, the primer-binding sites were similar in MHV-68 (ILK+: two mismatches, KG1+: three mismatches, TGV+: one mismatch, IGY+: one mismatch). We therefore tested whether it was capable of detecting MuHV-4. MuHV-4 (strain MHV-68) in a BAC vector (provided by B. Dutia, The Roslin Institute, Edinburgh) was tested in triplicate at dilutions of 0.5 and 1 ng µl^−1^, and was detected by this PCR in all cases. Thus if MuHV-4 was present in these wild wood mice, it should have been detectable both with the degenerate and non-degenerate primers used here.

#### Statistical analyses.

All statistical analyses were performed in R, v. 2.13.2 (R Development Core Team, 2011) and considered wood mice only. Seroprevalence in bank voles, which was assessed systematically in 2010 only, was too low for statistical analysis (see Results). We first tested the extent of agreement between IFA- and PCR-based detection of WMHV across samples to assess each assay’s sensitivity and ability to represent true infection status. To test whether seropositivity in young animals may be driven by maternal antibodies, we investigated the relationship between host age and seropositivity.

Ecological predictors of WMHV infection were explored using binomial GLMs, using a consensus measure of infection (a mouse positive by either IFA, PCR or both). Since we found clear evidence for maternal antibodies (see Results), the main analyses of WMHV infection predictors were repeated for adult mice only, in which maternal antibodies should be negligible. Since mice were often represented multiple times within this dataset, to control for the influence of pseudoreplication, we randomly selected one data point per individual for analysis. We first created a full model including all terms and interactions of interest. Main effects considered were month (as a factor), host age (as a three-level factor: juvenile, subadult, adult), body mass (as a covariate) and trapping grid (as a five-level factor). Since reproductive activity manifests differently in males and females, we used a five-level factor to test for effects of reproductive status and sex combined (reproductive status/sex: abdominal, descended, scrotal, pregnant or perforated, not pregnant or perforated). Since it has been suggested that herpesviruses may be vector-borne (Ficová *et al.*, 2011), we included terms indicating whether either ticks or fleas were detected on the mouse. Interactions between month and year (to allow seasonal patterns to vary between the two years studied), and reproductive status and body mass (to allow the effect of reproductive status to vary with body mass) were also included. Full models were simplified by backwards, stepwise elimination of non-significant terms (*P*>0.10), starting with interactions, to obtain a minimal model with all terms *P*<0.05. In addition to this cross-sectional analysis, we also explored factors predicting the transition of individuals from being WMHV negative to positive (according to the consensus measure of IFA and PCR); for each mouse negative at first capture (time, *t*), we tested whether the set of variables outlined above predicted the acquisition of infection by the following month (time, *t*+1), using a binomial GLM.

To assess the effect of WMHV infection on host survival, we used a quasi-Poisson GLM to test whether the duration of recapture (the number of months between first and last capture within a year) was predicted by WMHV infection at first capture. We included the month of first capture as a covariate (to control for the fact that animals caught earlier in the year had more opportunity to be recaptured), reproductive status/sex, grid, and interactions between year and both WMHV status and month. This analysis was limited to mice first captured as adults, to minimize any potential influence of maternal antibodies. It also excluded mice first captured in December (which have no chance of recapture within that year) and in May 2010, so that a month*year interaction could be fitted (mice were not trapped in May 2009).
